# Association between Rash and a Positive Drug Response Associated with Vinorelbine in a Patient with Primary Peritoneal Carcinoma

**DOI:** 10.1155/2013/825717

**Published:** 2013-09-01

**Authors:** Mustafa M. Mohammad, Kostas N. Syrigos, M. Wasif Saif

**Affiliations:** ^1^Tufts University School of Medicine, 136 Harrison Avenue, Boston, MA 02110, USA; ^2^Department of Oncology, Sotiria General Hospital, Athens, Greece; ^3^Section of GI Cancers and Experimental Therapeutics, Tufts University School of Medicine, 800 Washington Street, Boston, MA 02111, USA

## Abstract

Vinorelbine (Navelbine, VRL) is commonly used for platinum-resistant ovarian cancer and has been shown to be effective in patients with recurrent primary peritoneal carcinoma. Of VRL's major side effects, skin rash is uncommon, and, if it does occur, it is usually localized to site of injection. In this case report, a 71-year-old Hispanic female with primary peritoneal carcinoma received single agent VRL as fourth-line regimen, which she tolerated very well except for a skin rash related to VRL. The rash continued to progress throughout 6 cycles of VRL, and follow-up CT/PET scan demonstrated complete metabolic and radiological responses. We, therefore, believe that this rash was linked to VRL administration and correlated with response to therapy. Rash has been recognized as a useful surrogate marker with targeted agents such as cetuximab and erlotinib; to the best of our knowledge, this case report describes the first patient with a possible drug rash and its association with a positive outcome. This case report incites interest in further investigation of similar cases to support this observation, since there is a lack of reports of skin rash with VRL therapy.

## 1. Introduction

Vinorelbine (Navelbine, VRL), topotecan, and gemcitabine all have shown activity in platinum-resistant ovarian cancer [1–4]. Granulocytopenia, leukopenia, anemia, asthenia, nausea, vomiting, and constipation constitute the most common side effects associated with VRL. Although vinca alkaloids are vesicants, most dermatologic toxicities are restricted to injection sites, and unspecified rash has been rarely described with VRL [5]. In the phase II study, approximately 13% of patients demonstrated some sort of dermatologic toxicity [1]. In 3 clinical studies of patients with nonsmall cell lung cancer and advanced breast cancer, patients were treated with single agent VRL on a dosing schedule of 30 mg/m^2^ of VRL weekly. 28% of patients demonstrated injection site reactions, with only 2% being grade 3 [5]. Tables 1 and 2 depict the incidence of hematological versus nonhematological toxicities associated with VRL.

## 2. Case Report

A 71-year-old Hispanic female with a past medical history significant for diabetes mellitus presented to her primary care physician with a two-month history of fatigue, weight loss, early satiety, and new onset of left neck swelling. A CT scan of the neck and chest showed left-sided cervical and mediastinal lymphadenopathy. In addition, bulky lymphadenopathy of the upper abdomen surrounding the aorta and inferior vena cava was noted. Subsequently, a PET scan showed multiple masses, including a 10 × 5.6 × 7.8 cm mass between the spleen and stomach, a 7.2 × 5.2 cm mass near the ascending colon, and a 5.8 × 4.5 cm right adrenal mass. Her CA 125 level at the time of presentation was 24,800 U/mL. She also had an elevated CA 15.3 and normal CA 19.9 and CEA levels. She had a normal mammogram and a colonoscopy two years priorly without any evidence of disease. She had a total abdominal hysterectomy and bilateral salpingooophorectomy 13 years prior to presentation. A fine needle aspiration of one of the abdominal masses was positive for adenocarcinoma. Immunohistochemistry was positive for CK19, CK7, BerEP4, and WT1 and negative for calretinin, CD20, CDK2, TTF1, and ER/PR. Based on these findings, in addition to the clinical features, laboratory data, and radiographic imaging, a diagnosis of primary peritoneal carcinoma was made.

**Figure 1:**
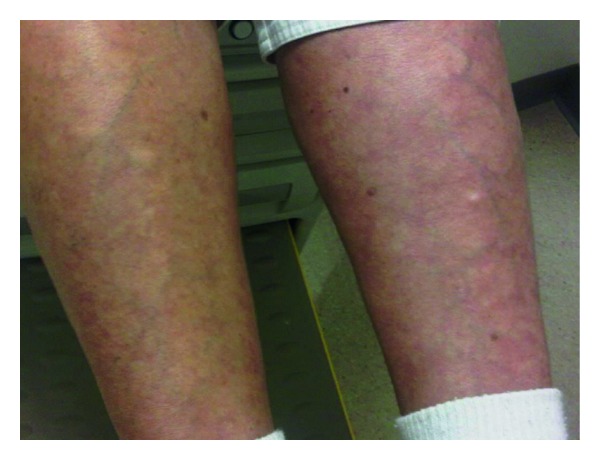
Rash on lower extremities.

She was started on carboplatin, docetaxel, and erlotinib. She received 11 cycles of this regimen, and it was discontinued because of docetaxel-induced fluid retention syndrome. After 3 months of this regimen, a follow-up CT scan showed a 41% reduction in disease burden per RECIST criteria. Her regimen was then switched to bevacizumab because of radiographic progression of disease in the lungs. She received 5 cycles of bevacizumab before discontinuing this regimen secondary to uncontrolled hypertension despite antihypertensives. Gemcitabine was given briefly, but it was discontinued secondary to fluid retention. She was transitioned to single agent VRL every 2 weeks. During this time, patient developed an erythematous, maculopapular rash on her anterior lower extremities, with minimal itch (Figure 1). Concerns for deep vein thrombosis (DVT) versus dermatitis were raised. Venous Doppler of both legs was performed, and no DVT was found. There was no clinical evidence of dermatitis after consultation with a dermatologist. No patch test was performed. Rash was more localized on legs, left greater than right. Rash was managed with local steroid cream and Claritin PO. Rash improved in 2 weeks with less urticaria and redness, and VRL was continued. After 6 cycles of VRL, the patient demonstrated that she had complete metabolic and radiological response, without evidence of mediastinal, hilar, or abdominal lymphadenopathy. Due to complete response, she was taken off of VRL and continues to be disease-free 3 years since diagnosis with normal tumor marker values.

## 3. Discussion

VRL is a semisynthetic vinca alkaloid that interferes with microtubule assembly. Vinca alkaloids consist of 2 multiringed units, vindoline and catharanthine. VRL differs from other vinca alkaloids in that the catharanthine is site where structural modification occurs. VRL is commonly used for nonsmall cell lung cancer, advanced breast cancer, platinum-resistant ovarian cancer, and primary peritoneal carcinoma, and has been shown to be an effective treatment.

A phase II study with combination of topotecan and VRL demonstrated a response rate of 44% in patients with platinum-resistant primary peritoneal or ovarian cancer. This study showed median time to progression (TTP) of 4.37 months, with median overall survival (OS) of 16.4 months [1]. In another phase II study, VRL demonstrated a 21% response rate with a median TTP of 3.1 months and median OS of 10.1 months [2]. Yet in another phase II study of single agent VRL given weekly at a dose of 25 mg/m^2^ given to patients with platinum-resistant ovarian cancer, VRL demonstrated a response rate of 21%. Median OS was 10 months [3]. In the randomized phase III trial of gemcitabine, gemcitabine demonstrated an overall response rate of 6.1% with a median TTP of 3.6 months and median OS of 12.7 months [4]. Though results are modest in all three trials, they are comparable with other second-line treatments for platinum-resistant ovarian cancer. Based on these studies, VRL was a suitable fourth-line medication for the patient.

Granulocytopenia is consistently VRL's major dose-limiting toxicity, although it was often reversible and noncumulative. Of note, there has been no previously reported association between rash development and a positive drug response which was previously reported in trials of VRL.

A study by eHealthMe studied the relationship of VRL and rash in 1,314 VRL users from 28 FDA reports. Only 27 patients (2.05%) demonstrated rash with 66% experiencing the rash within 1 month and 33% within 1–6 months. No patient developed rash after 6 months of treatment with VRL. Notably, the study did not separate patients on single agent VRL therapy versus combination therapy [6].

Rash has been recognized as a useful surrogate marker with targeted agents such as cetuximab and erlotinib, among others.

In summary, we believe that this patient is the first report of such an association between rash and response to VRL in a patient with primary peritoneal carcinoma. This case report incites interest in further investigation of similar cases to support this observation, since there is a lack of reports of skin rash with VRL therapy. We would be interested in finding out if anyone from the giant group of oncologists who read this journal has experienced a similar coincidence of a remarkable response to the therapy and dermatological side effects.

## Figures and Tables

**Table 1:** Summary of hematological events in 365 patients receiving single agent VRL^∗†^.

Adverse event	All patients (*n* = 365)	NSCLC (*n* = 143)
Bone marrow		
Granulocytopenia		
<2,000 cells/mm^3^	90%	80%
<500 cells/mm^3^	36%	29%
Leukopenia		
<4,000 cells/mm^3^	92%	81%
<1,000 cells/mm^3^	15%	12%
Thrombocytopenia		
<100,000 cells/mm^3^	5%	4%
<50,000 cells/mm^3^	1%	1%
Anemia		
<11 g/dL	83%	77%
<8 g/dL	9%	1%
Hospitalizations due to granulocytopenic complications	9%	8%

*None of the reported toxicities were influenced by age. Grade based on modified criteria from the National Cancer Institute.

^†^Patients with NSCLC had not received prior chemotherapy. The majority of the remaining patients had received prior chemotherapy

(http://www.fda.gov/ohrms/dockets/ac/04/briefing/4021b1_10_vinorelbine%20label.pdf).

**Table 2:** Summary of nonhematological adverse events in 365 patients receiving single agent VRL^∗†^.

Adverse event	All grades		Grade 3		Grade 4	
	All patients	NSCLC	All patients	NSCLC	All patients	NSCLC
Clinical chemistry elevations						
Total bilirubin (*n* = 351)	13%	9%	4%	3%	3%	2%
SGOT (*n* = 346)	67%	54%	5%	2%	1%	1%
General						
Asthenia	36%	27%	7%	5%	0%	0%
Injection site reactions	28%	38%	2%	5%	0%	0%
Injection site pain	16%	13%	2%	1%	0%	0%
Phlebitis	7%	10%	<1%	1%	0%	0%
Digestive						
Nausea	44%	34%	2%	1%	0%	0%
Vomiting	20%	15%	2%	1%	0%	0%
Constipation	35%	29%	3%	2%	0%	0%
Diarrhea	17%	13%	1%	1%	0%	0%
Peripheral neuropathy^‡^	25%	20%	1%	1%	<1%	0%
Dyspnea	7%	3%	2%	2%	1%	0%
Alopecia	12%	12%	≤1%	1%	0%	0%

*None of the reported toxicities were influenced by age. Grade based on modified criteria from the National Cancer Institute.

^†^Patients with NSCLC had not received prior chemotherapy. The majority of the remaining patients had received prior chemotherapy.

^‡^Incidence of paresthesia plus hypesthesia (http://www.fda.gov/ohrms/dockets/ac/04/briefing/4021b1_10_vinorelbine%20label.pdf).
